# Software Tool for Visualization and Validation of Protein Turnover Rates Using Heavy Water Metabolic Labeling and LC-MS

**DOI:** 10.3390/ijms232314620

**Published:** 2022-11-23

**Authors:** Henock M. Deberneh, Rovshan G. Sadygov

**Affiliations:** Department of Biochemistry and Molecular Biology, The University of Texas Medical Branch, 301 University Blvd, Galveston, TX 77555, USA

**Keywords:** in vivo protein turnover, heavy water metabolic labeling, isotope distribution, time series of isotope labeling, graphical user interface for mass spectral data

## Abstract

Metabolic stable isotope labeling followed by liquid chromatography coupled with mass spectrometry (LC-MS) is a powerful tool for in vivo protein turnover studies of individual proteins on a large scale and with high throughput. Turnover rates of thousands of proteins from dozens of time course experiments are determined by data processing tools, which are essential components of the workflows for automated extraction of turnover rates. The development of sophisticated algorithms for estimating protein turnover has been emphasized. However, the visualization and annotation of the time series data are no less important. The visualization tools help to validate the quality of the model fits, their goodness-of-fit characteristics, mass spectral features of peptides, and consistency of peptide identifications, among others. Here, we describe a graphical user interface (GUI) to visualize the results from the protein turnover analysis tool, d2ome, which determines protein turnover rates from metabolic D_2_O labeling followed by LC-MS. We emphasize the specific features of the time series data and their visualization in the GUI. The time series data visualized by the GUI can be saved in JPEG format for storage and further dissemination.

## 1. Introduction

Cellular proteins are in a dynamic equilibrium. Protein concentrations are maintained while they are continuously synthesized and degraded. The equilibria are tissue-specific, and they shift during organismal development, aging, and diseases. Metabolic stable isotope labeling followed by liquid chromatography and mass spectrometry (LC-MS) has been a powerful tool to study in vivo protein turnover on a large scale and high throughput [1,2]. As a labeling agent, heavy water (drinking water enriched in D_2_O) is easy to use, cost-efficient, and does not require adaptation period [3]. Low (<8%) concentrations of D_2_O enrichments are normally used in drinking water [4]. It results in the composite spectra of unlabeled and labeled forms of a peptide in MS1. Statistical [5] and analytical [6] approaches to de-convolve the spectra have been described. A recent study [7] revealed that the precursor enrichment in D_2_O labeling was nearly instantaneous, and a single exponential curve was sufficient for the modeling. In contrast, the precursor enrichment in heavy amino acid labeling [8] was delayed and tissue specific [7]. Therefore, the modeling of label incorporation in amino acid labeling was more complex and required more parameters.

Since the data are generated for thousands of proteins from tens of thousands of peptides at every time point of labeling, manual data processing is impractical. Several publicly available software tools [7,9,10] have been developed to process the mass spectral data and database search results to automate protein turnover rate estimations. The turnover rates are obtained from the exponential decay modeling of the monoisotopic relative isotope abundance (RIA). The tools generate protein turnover rates and goodness-of-fit (GOF) measures of the model, such as coefficient of determination (R^2^), Pearson correlation, and standard deviation, among others. The results are normally reported in tables and saved in output files formatted in csv format. Though the turnover estimation tools automate data processing, the visualization and annotation of the results are as important. The csv formatted files can be read and processed using scripts in R [11] or Phyton environments. However, it requires familiarity with these environments. Therefore, a graphical user interface (GUI) to enter input data, easily access results for each protein/peptide, and obtain information about the statistical GOF measures is important. A protein turnover estimation software tool [12] for metabolic labeling with a heavy amino acid (^13^C_6_-Lys) contained a GUI, ApplE Turnover, to facilitate the data analysis. Another tool for protein turnover estimation from [5,5,5-^2^H_3_] Lue labeled samples, TurnoveR [13], used the functions of Skyline [14], an MS data analysis platform. Here, we report on our implementation of a GUI for a software tool, d2ome [10], to estimate protein turnover rates from D_2_O labeling. The GUI facilitates several manually laborious steps in the data input, the selection of data processing parameters, and, importantly, it plots experimental time points and theoretical fit, shows the GOF measures, and spectral features (mass-to-charge ratio, *m*/*z*, the monoisotopic abundance, charge state, and the number of exchangeable hydrogens) of the peptide and its amino acid sequence. Every protein can be located by an easy search or from a drop-down list of alphabetically sorted protein names. mzML [15] (mass spectral) and mzid [16] (database search results) files are automatically matched in the input. Considering that the data for protein turnover is highly voluminous, the GUI will facilitate the data analysis, visualization, and validation of the results.

## 2. Results and Discussions

**Figure 1:**
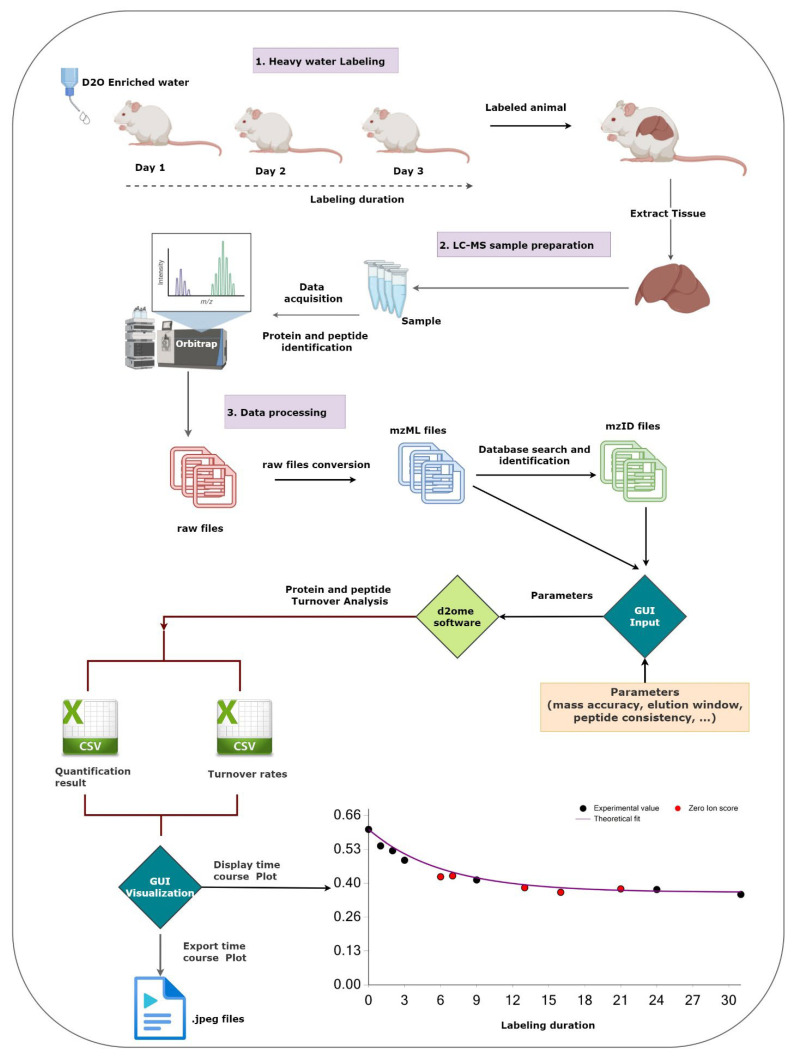
The figure shows the workflow of the main experimental and data processing of protein turnover studies and the role of the graphical user interface (GUI). The GUI aids in setting up the protein turnover rate estimations (creating input data from mzML and mzid files) and visualizing the results to facilitate the validations.

The time series data used in protein turnover studies is more complex than the static proteomics data. Thus, in static proteomics, the proteome is normally characterized by peptide sequence and its post-translational modifications, abundance, chromatographic retention time, *m*/*z*, and charge state. In contrast, protein turnover data, in addition to the listed information, requires the number of exchangeable hydrogens, body water enrichment in deuterium, the number of experiments in which the peptide was quantified, GOF measures (R^2^, RMSE, SD) between the experimental data and the theoretical fit, the monoisotopic abundance, and the accuracy of the isotope distribution between the estimated and LC-MS data for the unlabeled sample. A typical workflow of experimental and data processing steps is shown in Figure 1.

### 2.1. Data Input and Data Processing Parameters

**Figure 2:**
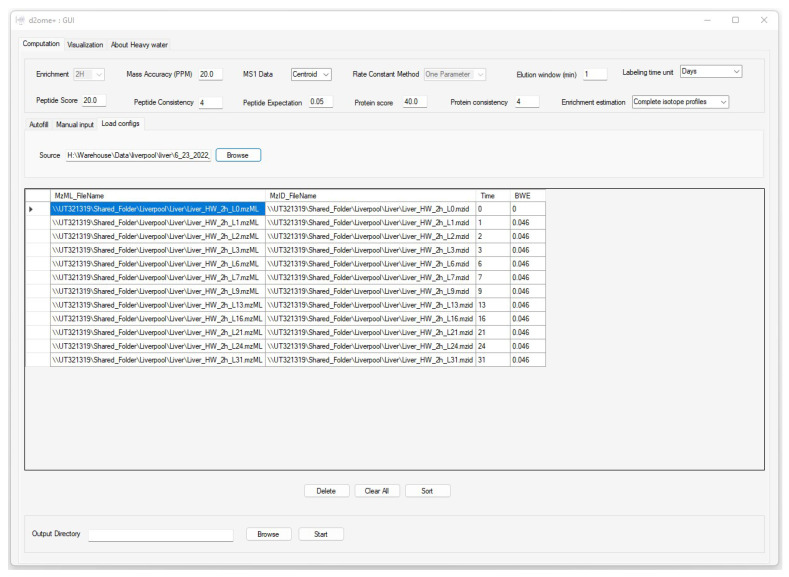
The graphical user interface (GUI) to input data and parameters for the protein turnover rate estimations. Time and BWE are the labeling duration and body water enrichment in deuterium for the corresponding mzML and mzid files (experiments).

Figure 2 presents a sample screenshot of the GUI for data and parameter input. The GUI enables users to input sets of database search results and corresponding mass spectral data, body water enrichment in deuterium, peptide and protein consistencies (the minimum number of experiments in which a peptide and a protein are identified in MS/MS), and the corresponding labeling duration in a tabular format. The software has a feature to automatically populate pairs of input files from the source folder by matching the file names. It allows users to reload all configurations from the previous runs for re-runs with different parameters. The GUI allows users to optimize the quantification results by customizing the input parameters. The parameters include spectral mass accuracy, retention time window for peak detection and integration, the threshold of peptide score (Mascot [17] Ion score), and expectation.

Consistent identifications of peptides and proteins from tandem mass spectra are essential in time series experiments. Since DDA is semi-stochastic in the selection of ions to be fragmented, we implemented a match between-runs (MBR) approach to enable the quantification of peptides that are missing in some experiments, but their features (chromatographic elution profile corresponding to *m*/*z* and charge state at the allowed chromatographic time elution window) are detectable. The approach implemented the retention time alignment strategy using raw mass spectral profiles [18]. The time window in which the missing peptide features will be searched is adjustable in the GUI. MBR increases proteome coverage across the labeling duration time series.

### 2.2. The Output of Data Processing

d2ome [10] computes turnover rates for proteins and peptides using the non-linear least squares regression on the monoisotopic RIA. It generates two main outputs: EntryName.RateConst.csv and EntryName.Quant.csv. The first entry in the file names is the Uniprot [19] entry name for a protein. The *.Quant.csv file contains comprehensive information about each peptide of a protein. Each peptide entry is a row of information that contains the amino acid sequence, the charge state of the precursor, theoretical *m*/*z* of the peptide sequence, theoretical isotope abundances (natural isotope abundances), precursor *m*/*z*, the highest Mascot Ion score, Mascot expectation, mass accuracy (in ppm), scan number, the integrated (from MS1 scans in LC-MS) abundance of the mass isotopomers (six), elution start and end times that were used to calculate the isotopomer abundances, and the monoisotopic peak width in the mass-to-charge domain (used only for data in profile mode).

The rows of *.RateConst.csv file of a protein contain: the peptide sequence, its uniqueness (distinct or shared with other proteins), peptide rate constant and corresponding confidence intervals, the correlation value between theoretical fit and experimental data, RMSE, the absolute deviation between the theoretical and experimental isotope profiles (before the start of labeling), peptide charge, sequence *m*/*z*, the number of accessible hydrogens (N_EH_), the number of data points (NDP), R^2^ of the theoretical fit, and the average abundance of the monoisotope.

### 2.3. Visualization of the Results

**Figure 3:**
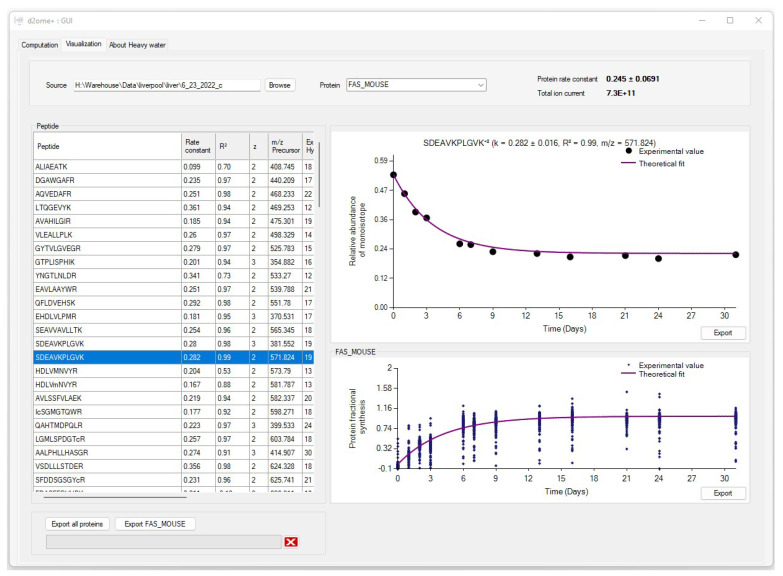
The graphical user interface (GUI) for the results of protein turnover rate estimations. Results for each protein can be accessed by name search. For every peptide of a protein, the experimental time series data and its theoretical fit can be visually examined.

The visualization tab of d2ome has two main charts that depict the time series [20] data used for peptides and protein degradation rate computation, Figure 3. It provides easy access to turnover rate estimation results for each protein. For every protein peptide, the monoisotopic RIAs estimated from the isotope profiles in comparison with the theoretical fit can be visualized. This approach visualizes the correspondence between the experimental points and the expected theoretical values, which are computed based on the degradation rate constant.

**Figure 4:**
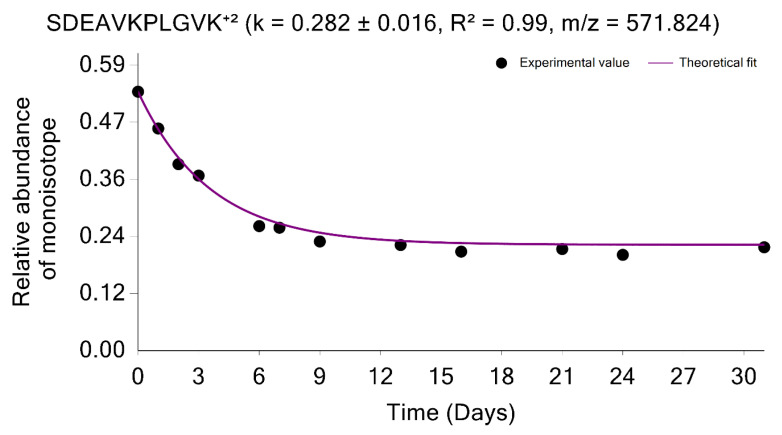
The graphical user interface enables comprehensive visualization of the results of protein turnover studies from metabolic D_2_O labeling and LC-MS experiments. Time series of monoisotopic RIAs (y-axis) are shown along the labeling duration (*x*-axis). The solid line shows the fit from the computed degradation constant for SDEAVKPLGVK⁺^2^ peptide from FAS_MOUSE protein.

**Figure 5:**
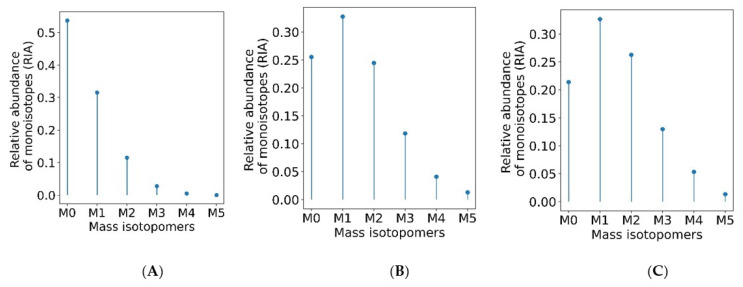
The monoisotopic RIA depletes with the labeling duration. Isotope profiles of SDEAVKPLGVK⁺^2^ peptide (**A**) from an unlabeled sample (**B**) from a labeled sample (day 7) (**C**) from a labeled sample (day 31).

The estimation of the monoisotopic RIA requires accurate measurements of the abundances of all mass isotopomers of a peptide. Since mammalian samples are complex, peptide species often co-elude and interfere with the mass profile of the target peptide. This GUI enables users to graphically validate the quality of experimental input data (the time series of monoisotopic RIA) in comparison with the theoretical fit. Figure 4 shows the monoisotopic RIAs estimated from the isotope profiles in comparison with the theoretical fit for the peptide sequence, SDEAVKPLGVK⁺^2^ from FAS_MOUSE protein. For this protein, the experimental isotope distributions of each peptide at every time of labeling are in the FAS_MOUSE.Quant.csv file. The unlabeled and labeled [7] (7 and 31 days) isotope profiles of the SDEAVKPLGVK⁺^2^ peptide are presented in Figure 5. The monoisotopic RIA was computed as the ratio of monoisotopic abundance to the sum of abundances of all mass isotopomers. The data can be used as additional validation of the label incorporation.

**Figure 6:**
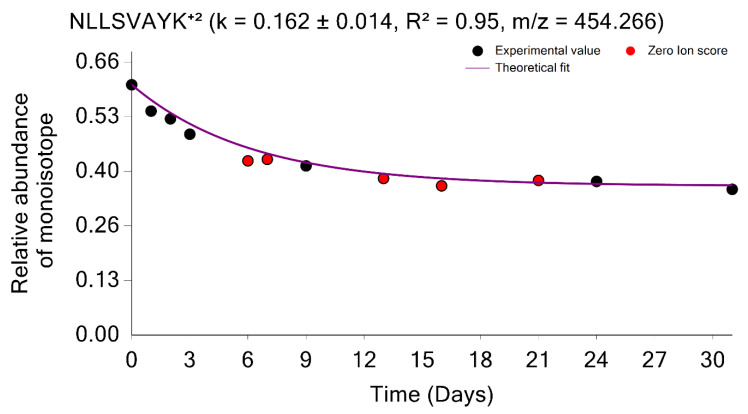
Time course plot of monoisotopic RIAs for NLLSVAYK⁺^2^ peptide from the 1433B_MOUSE protein. The experimental time points quantified using the match between runs are shown in red.

As mentioned above, the quantifications using MBR transfers are important in stable isotope labeling experiments. The MBR procedure may result in false positive transfers [21]. The GUI provides the opportunity to examine the quality of the label incorporation estimation from the data obtained by using the MBR. Thus, the labeling time points, which were quantified using MBR, can be shown in red; it is demonstrated in Figure 6 for the peptide sequence NLLSVAYK⁺^2^ from the 1433B_MOUSE protein. Shown in red are the labeling time points (experiments) in which the peptide was not identified from an MS/MS spectrum. Instead, the quantification was performed based on the MBR. The use of MBR increases proteome coverage across the labeling time points. It is helpful to visually examine the MBR quantified time points, and the GUI provides this opportunity.

**Figure 7:**
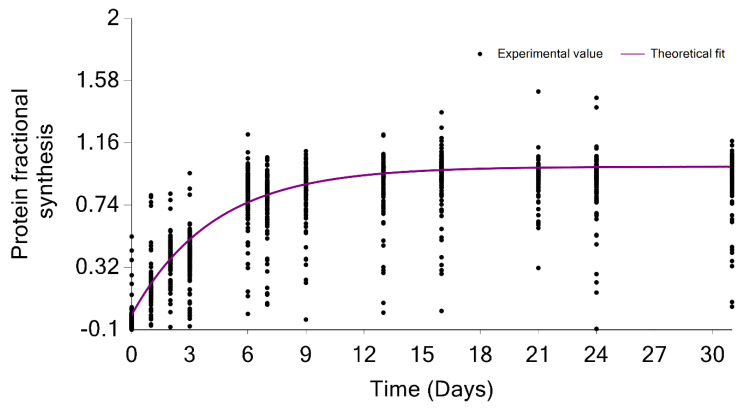
Protein turnover rate is computed from the median of the degradation constant of quantified peptides. The scatter plot indicates the fractional value of peptides in the FAS_MOUSE protein. The solid line shows the fit from the computed turnover rate for the protein.

The GUI also graphically shows the overall label incorporation from all peptides of a protein. It is illustrated in the time series of the FS. Figure 7 shows the protein FS in comparison with the theoretical fit for the FAS_MOUSE protein. The graph presents each peptide’s experimental FS as a scatter plot and the theoretical fit based on the protein rate constant as a solid line. Both figures can be exported as high-quality JPEG images. Furthermore, the software enables users to export charts separately or in a batch mode for all identified proteins and peptides. The GUI is a user-friendly application that makes searching and visualizing all proteins and peptides simpler. Users can easily switch between the visualizations of different proteins/peptides.

The visualization window also contains comprehensive information about each peptide of a protein in a tabular format. Each peptide entry is a row of information that contains the amino acid sequence, the charge state of the precursor, the theoretical *m*/*z* of the peptide sequence, the correlation between theoretical fit and experimental time series, RMSE, the absolute deviation between the theoretical and experimental isotope profiles, N_EH_, NDP, R^2^ of the theoretical fit, and the monoisotopic average abundance.

The currently available software tools for protein turnover studies from LC-MS-MS/MS data of deuterium-labeled samples (such as DeuterRator [9] and Riana [7]) simplify data analyses also by means of a GUI component. The GUI in DeuteRator [9] simplifies data entry and parameter selection. It plots and saves the FS time series and its theoretical fit for each protein. Output from Riana can be visualized in the R environment using supplied scripts. Our approach to the GUI development was motivated by that of ApplE Turnover [12]. A user can search for each protein, plot the experimental time series and theoretical fit of the monoisotopic RIA for every peptide, display the experimental time series and corresponding theoretical fit for the FS of a protein, export all figures, and view several GOFs of each peptide of a protein. It is possible to review previously processed results. We believe the GUI features address user needs in many cases. Our main goal in developing the GUI was to facilitate the visualization, quality assessment, validation, and dissemination of the turnover rate estimation.

### 2.4. Future Plans

The GUI developed in this work provides visualization of the theoretical fit to the experimental data points, the GOF measures, and spectral features for a peptide. An additional element for visual validation is the experimental isotope distribution of the peptide at the apex of its elution. This visualization of the distribution would allow us to validate the quality of the monoisotopic RIA estimation. We plan to implement this interactive feature in a future iteration of the GUI. Currently, the GUI interfaces with the database search output from Mascot. We intend to include support for other search engines.

## 3. Methods

In data modeling from metabolic D_2_O labeling and LC-MS experiments, the peptide/protein turnover rate is estimated by exponential decay modeling of the time course of the depletion of the monoisotopic RIA, I_0_(t), with the labeling duration, t:

(1) $$I_{0}\left(t\right)={\;I}_{0}^{\mathrm{asymp}}+\left(I_{0}\left(0\right)-{\;I}_{0}^{\mathrm{asymp}}\right)e^{-\mathrm{kt}}$$

where I_0_(0) is the monoisotopic RIA of an unlabeled peptide, I_0_^asymp^ is the monoisotopic RIA at the plateau of labeling, and k is the turnover rate (degradation rate constant) of a peptide. I_0_^asymp^ is obtained from the body water enrichment in deuterium (p_W_) and the number of hydrogens accessible to deuteriums in the water (N_EH_):

$${\;I}_{0}^{\mathrm{asymp}}=I_{0}\left(0\right){\left(1-\frac{p_{w}}{1-p_{H}}\right)}^{N_{\mathrm{EH}}}$$

The turnover rate is obtained from the non-linear regression of the experimental time series data of I_0_(t) on the theoretical decay function in Equation (1). The modeling is central to the turnover rate estimation. The GUI depicts experimental time points and the theoretical curve resulting from the regression for every peptide.

Another property used for the analyses of protein turnover is the fractional synthesis. For every peptide, the fractional synthesis (FS) is defined as:

(2) $$\mathrm{FS}\left(t\right)=\frac{I_{0}\left(0\right)-I_{0}\left(t\right)}{I_{0}\left(0\right)-{\;I}_{0}^{\mathrm{asymp}}}=1-e^{-\mathrm{kt}}$$

In Equation (2), the explicit dependency on the number of exchangeable hydrogens and natural monoisotopic RIA, which are characteristics of each peptide, are removed. The GUI depicts the FSs of all peptides of protein in a single figure.

The GUI in d2ome is a Windows Forms application developed in C# programming language. Windows Forms is a .Net Framework GUI library that provides an interface to develop multipurpose applications. It is composed of controls such as combo boxes, buttons, labels, list boxes, charts, and containers such as panels, group boxes, and others. In the course of the development of the GUI for the d2ome software tool, we had used a tab layout to switch between computation and visualization windows, a data grid view to display detailed peptide information in a tabular format, charts to display peptides time course data, and buttons to execute tasks such as loading data, searching proteins, exporting charts, and others. The GUI can be initiated either from the command line or from the application icon.

The GUI interfaces with d2ome in two stages, Figure 1. In the first stage, the GUI automates data input (matching pairs of mzML [22] and mzid files, body water enrichment, and labeling time course) and the specification of parameters (mass accuracy, the required number of labeling time points, database search scores, etc.). There is no limitation on the number of experiments (input files for processing). The GUI uses the experimental data and parameters to create an input set for d2ome to compute peptide/protein turnover rates. d2ome writes out the results for every protein in .Quant.csv and .RateConts.csv files. The .Quant.csv file of a protein contains information about the amino acid sequence, theoretical isotope distribution of unlabeled peptide, *m*/*z*, charge, scan number of MS/MS identification, mass accuracy, database search score, and mass isotopomer abundances (M_0_-M_5_) from each experiment for every identified and quantified peptide of the protein. The .Rate.Const.csv file of a protein contains the results of rate constant (turnover rate) calculations for every peptide, GOF to the theoretical model, Equation (1), and statistical properties of the computed rate constant, such as standard deviation (SD), root-mean-squared-error (RMSE), coefficient of determination (R^2^), Pearson correlation, averaged (from all experiments) monoisotopic abundance, and protein turnover rate. Normalized (by the median of medians of base peak abundances from each experiment) protein abundance is also reported in the file. These data are depicted by the GUI in the second stage of interfacing with d2ome. All figures (experimental time series and its theoretical fit, FS for a protein) can be exported as high-quality JPEG images. Furthermore, the software enables users to export charts separately or as a batch process for all identified proteins and peptides. The tool is available in the GitHub repository, https://github.com/rgsadygov/d2ome (accessed on 17 November 2022).

### Data Used in This Work

The figures and examples shown in the paper were obtained from processing a publicly available data set of mouse liver proteome [7]. Labeling and LC-MS experiments are described in the original publication. In brief, adult male C57BL/6JOlaHsd mice were labeled with deuterium oxide. Murine liver tissues were collected at twelve labeling time points: 0, 1, 2, 3, 6, 7, 9, 13, 16, 21, 24, and 31 days. The body water enrichment in deuterium was determined to be 0.046 in all labeled samples. The mass spectral data were acquired in the data-dependent acquisition mode (DDA) using a Q-Exactive HF quadrupole-Orbitrap mass spectrometer. The raw mass spectral data are available on ProteomeXchange at accession PXD029639.

## 4. Conclusions

We developed a graphical user interface to facilitate the data analysis of protein turnover studies from time series data of metabolic labeling with D_2_O and LC-MS. The turnover rate calculations use a large number of experimental inputs (time series of label enrichment) and parameters (body water enrichment, mass accuracy, peptide/protein identification consistency, etc.). The GUI automates data input and parameter selection.

The validation of the protein turnover results requires information about various spectral features (*m*/*z*, the abundance of the monoisotopic RIA, NDP, etc.) and statistical measures of GOF (R^2^, Pearson correlation, SD, RMSE, etc.). The GUI depicts the theoretical fit to the experimental time series data, thus allowing a visual evaluation of the fit. The statistical measures of the model show the quality of the GOF, which also helps to estimate the quality of the theoretical fit. All generated figures for every peptide of a protein can be exported in JPEG format for further dissemination.

## Data Availability

Tools reported in this paper are available in the GitHub repository: https://github.com/rgsadygov/d2ome (accessed on 17 November 2022).

## Abbreviations

DDA—data-dependent acquisition; GOF—goodness of fit; GUI—graphical user interface; FS—fractional synthesis; LC-MS—liquid chromatography coupled with mass spectrometry; MBR—match between runs; NDP—the number of data points; N_EH_—the number of exchangeable hydrogens; RIA—relative isotope abundance; RMSE—root mean squared error; SD—standard deviation.
